# Effects of victimization and perpetration in observing bullying scenes: an eye-tracker study{es}: Efectos de la victimización y la perpetración en la observación de escenas de acoso: un estudio con eye-tracker

**DOI:** 10.1016/j.ijchp.2024.100451

**Published:** 2024-02-28

**Authors:** Laura Menabò, Simona C.S. Caravita, Grace Skrzypiec, Phillip Slee, Annalisa Guarini

**Affiliations:** a Department of Psychology “Renzo Canestrari”, University of Bologna, Viale Carlo Berti Pichat, 5, 40127, Bologna, Italy; b Faculty of Arts and Education Norwegian Centre for Learning Environment and Behavioral Research in Education, University of Stavanger, Kjell Arholms gate 41, 4021 Stavanger, Norway; c Department of Education, Flinders University, 182 Victoria Square, 5000 Adelaide, Australia

**Keywords:** Bullying, Roles, Attention allocation, Eye-tracker, Bully/victims

## Abstract

**Introduction:**

Previous research showed that bullying experiences are associated with different ways of interpreting and behaving in bullying dynamics. However, it remains uncertain whether these distinctions can already be present during the first step of information processing: the allocation of attention.

**Aims:**

The study explored attentional patterns of Italian students with different bullying experiences in daily life while observing different roles represented through bullying vignettes.

**Methods:**

Participants (72 students, *M*_age_= 11.18) were categorized as victims, bully-victims, or not involved based on their scores on a self-report questionnaire. They observed 9 bullying vignettes on which different portraits were presented (bully, victim, pro-bully, defender, bystander) while the eye-tracker registered attentional indexes (fixation, visit and duration).

**Results:**

Kruskal- Wallis and pairwise comparisons revealed a significant effect for the portraits of the bully and the pro-bully as bully-victims exhibited greater fixations and visits than victims, while students not involved showed no significant differences with the other groups.

**Conclusion:**

Our research reveals that bully-victims focused more on threatening cues while victims diverged their gaze from them, confirming that the experience of bullying influences how they explore aggressive situations. Learning how involved students direct their attention helps us understand different responses, leading to powerful interventions.

Bullying represents a pervasive and public health issue that affects students across various age groups and socio-cultural contexts (Biswas et al., 2020). Although the definition of bullying remains a subject of ongoing debate (Hellström, Thornberg, & Espelage, 2021), it is usually recognized for the repetition over time, the imbalance of power between the perpetrator(s) and the victim(s), and the intentionality to inflict harm (Olweus, 1992).

Perpetrators (or bullies) are individuals who consistently and purposefully target others with the intent to harm without themselves being subjected to aggression. Conversely, victims are those students who suffer repeated attacks over an extended period, often characterized as defenselessness and withdrawal (see Guzman‐Holst & Bowes, 2021). Another distinct group comprises students engaged in both perpetrating bullying and experiencing victimization, commonly referred to as "bully-victims" (Husky et al., 2020; Walters, 2020).

Although it is still not clear why some students become bully-victims (Malamut & Salmivalli, 2021), previous studies showed that traits like reduced empathy, limited prosocial behavior, fragile family bonds, or difficulties in anger regulation could contribute to their profile (Chan & Wong, 2015; Espino, Guarini, Menabò, & Del Rey, 2023). Bully-victims are the most disliked group, and they are more likely to have higher levels of depression and self-harm compared to the other groups (Georgiou & Stavrinides, 2008).

Although much progress has been made in the study of bullying, many gaps remain to be clarified (e.g., Gini, Pozzoli, Jenkins, & Demaray, 2021). Specifically, previous studies highlighted differences in how bullies, victims, bully-victims, and those not involved consider and react to bullying situations (e.g., Camodeca, Goossens, Schuengel, & Terwogt, 2003). One explanation offered for this difference by Camodeca et al. (2003) is based on the Social Information Processing theory (SIP, Crick & Dodge, 1994).

SIP asserts that people undergo a sequence of cognitive steps that influence how they behave in social situations. Briefly, these steps involve 1) encoding social cues, 2) interpreting the cues, 3) clarifying goals, 4) constructing a response, 5) making a decision on the response and 6) enacting the behavioral response (Crick & Dodge, 1994). In particular, the first step holds significant importance, as any inaccuracies in encoding during this phase can have a ripple effect, influencing all subsequent steps and potentially leading to maladaptive behaviors (e.g., Garon, Lavallee, Estay, & Beauchamp, 2018). Despite its crucial role this aspect remains an underexplored area of research in the bullying context (Kellij, Lodder, Van Den Bedem, Güroğlu, & Veenstra, 2022; Troop-Gordon et al., 2019).

In the present study, our focus was on encoding social cues, which involves directing attention to relevant social stimuli. This process is automatic, fast and selective, guiding us to identify pertinent information within our environment (Horsley, de Castro, & Van der Schoot, 2010). In particular, we utilized eye-tracking technology to investigate the attentional patterns of students involved as victims, bullies, bully-victims, and those not involved, while observing various scenes of bullying.

The scenes used in this study highlighted the group dynamics inherent in bullying, presenting five distinct portrayals of individuals who act in specific roles already outlined in the literature (Salmivalli, Lagerspetz, Björkqvist, Österman, & Kaukiainen, 1996). Indeed, beyond the bully and the victim, one role is the "pro-bully," which is comprised of individuals who actively engage in aggressive behaviors or support the bully through laughter and encouragement. On the opposite end, the "defender" stands up for the victim, protecting and supporting them. Additionally, the "passive bystander" merely observes the bullying scenes without intervening or taking any action (Pouwels, van Noorden, Lansu, & Cillessen, 2018; Salmivalli et al., 1996).

Examining how students with diverse experiences in bullying (as victims, bullies, and bully-victims) focus their attention to salient social cues in bullying situations can provide valuable insights. This understanding is crucial for comprehending behavioral responses and designing effective interventions.

## Social Information Processing Theory and Aggression

Over the years, SIP has primarily been employed to study aggressive behaviors in children and adolescents. In this context, numerous studies have consistently shown a connection between various steps of SIP and aggressive behavior (Coy, Speltz, DeKlyen, & Jones, 2001; Dodge, Bates, & Pettit, 1990; Lochman & Dodge, 1994; Matthys, Cuperus, & Engeland, 1999) as further supported by a meta-analysis (De Castro, Veerman, Koops, Bosch, & Monshouwer, 2002).

However, while evaluating the later steps of SIP is relatively straightforward, assessing the first step (encoding social cues) poses a complex challenge due to its automatic and rapid nature (Van Nieuwenhuijzen et al., 2017). Earlier research attempted to evaluate encoding through methods such as recalling vignettes or scenarios (e.g., Dodge et al., 1990) or utilizing reaction time tasks (e.g., Gouze, 1987). For instance, Dodge et al. (1990) presented 24 vignettes depicting negative events to young boys and then asked them to recall the information. Aggressive children recalled fewer details for non-hostile cues compared to hostile cues, suggesting increased attention to hostile stimuli. An earlier study by Gouze (1987) utilized a reaction time task, where children were instructed to watch puppet shows depicting aggressive or non-aggressive situations. Their task was to promptly turn off a periodically illuminated light during the shows. The reaction time to turn off the light served as an indicator of the difficulty in shifting attention away from the puppet shows. The findings revealed that children with aggressive tendencies took more time to turn off the light when exposed to aggressive cues, suggesting a heightened inclination to focus on hostile stimuli.

However, it is important to note that while these studies provided valuable insights, they did not directly investigate encoding biases in attention allocation. Instead, they focused on processing derivatives, potentially influenced by various factors available for recall and verbal expression (Horsley et al., 2010). To address these challenges, limited research has examined attention allocation to visual stimuli depicting social situations through the use of eye-tracking technology. In one experiment by Laue et al. (2018), children were exposed to a common scenario in which a student spilled a glass of water on another student. This scenario was presented under various conditions: hostile, non-hostile, and ambiguous. The findings indicated that aggressive students spent significantly more time observing the scenario presented in a hostile condition compared to the non-hostile and ambiguous conditions

## Understanding Social Information Processing in Bullying Contexts

Although the SIP model provides a useful framework for understanding bullying behaviors, its application remains predominantly anchored to the later steps (Kellij et al., 2022). In this regard, Ziv, Leibovich, and Schechtman (2013) specifically focused on the second step and studied how students interpreted cues. They exposed participants to scenarios involving aggression and asked about their expectations regarding the outcomes through multiple questions. Victims tended to avoid social situations since they were anticipating hostility, while bullies and bully-victims perceived others as purposefully hostile and sought retaliation. On the other hand, students not involved perceived social situations as non-hostile and more likely to end positively. In an earlier study, Camodeca et al. (2003) analyzed how individuals categorized as bullies, victims, bully-victims, and students not involved processed the interpretation of social information. Both victims and bullies interpreted ambiguous social scenarios as hostile, exhibiting high levels of reactive aggression. However, only bullies reported high levels of proactive aggression. In the case of bully-victims, no differences from non-involved students were found in responding to hostile peer scenarios. Still, they resembled bullies in their responses to ambiguous social interactions, attributing more hostile intents and opting for proactive aggressive responses. In a more recent study, Mazzone, Yanagida, Camodeca, and Strohmeier (2021) pointed out a strict relationship among the second, third, and fourth steps of the SIP model. Indeed, students observed hypothetical ambiguous vignettes and the attribution of hostile intent was associated with the selection of antisocial goals, which in turn, was related to the generation of aggressive responses among participants with high levels of bullying.

Despite the relevance of the first step in the SIP theory, very few studies have analyzed encoding social cues in the context of bullying. Camodeca and Goossens (2005) investigated differences between bully, victim, pro-bully, defender, and bystander students in the initial three steps of SIP, by presenting different stories. Remarkably, no significant differences emerged in the encoding phase. By contrast, in the subsequent SIP step, both bullies and victims tended to attribute more hostile intentions to the perpetrator compared to their peers. The results also showed that while both bullies and victims exhibited reactive aggression, only bullies displayed proactive aggression.

Guy, Lee, and Wolke (2017) investigated how individuals classified as bully, bully-victims, and not involved engaged in the first and second steps of SIP, which involve the encoding and interpretation of social cues. Students were asked to identify emotions as part of the first step of the SIP. Subsequently, they were presented with both photographs and vignettes depicting social situations where the behavior could be interpreted as harmless or hostile. They found that in the first stage of emotion recognition, there were no discernible differences between victims and bullies. However, in the second step, victims and bully-victims displayed significant biases, particularly in their interpretations of hostility. They were more likely to attribute negative intentions to neutral stimuli compared to those not involved and bullies.

While these studies undoubtedly hold merit for highlighting the influence of bullying experiences on differences in various stages of SIP, three key issues emerge. Firstly, research focusing specifically on encoding in the context of bullying is quite scarce. Secondly, most of these studies on encoding rely heavily on recalling vignettes and scenarios or emotion recognition. However, as previously noted, vignettes and scenarios represent a process that can be verbally "explained", while encoding is an automatic and fast process. This distinction makes vignettes and scenarios more appropriate for examining later stages of SIP, such as interpretation. At the same time, emotion recognition entails a level of cognitive interpretation, as participants are required to reflect and interpret the emotions being displayed. This process does not fully capture the automaticity of the encoding process. Finally, the role of the bully-victim is further underrepresented in studies on encoding. To the best of our knowledge, only one study, conducted by Guy et al. (2017), has included bully-victims in the study of encoding, which raises concerns given this group's heightened risk of negative outcomes.

## Attention Allocation in Bullying Situations: The Use of Eye-Tracker

To gain a more comprehensive understanding of attention patterns in bullying situations, some researchers have taken a novel approach by directly assessing attention allocation using eye-tracking technology. This method allows for the direct measurement of attention allocation, bypassing the limitations of recalling vignettes or scenarios and offering a more accurate understanding of attention dynamics. According to a recent study by Menabò, Skrzypiec, Slee, & Guarini (2023), the results obtained with the eye-tracker are highly informative in understanding the allocation of attention when exploring bullying scenes, aligning well with verbal reports.

However, to our knowledge, only three studies have analyzed the effect of the experience of victimization and perpetration in daily life on encoding social cues. The first study by Caravita, Colombo, Stefanelli, and Zigliani (2016) utilized eye-tracking to explore differences in attention allocation in bullying and cyberbullying compared to prosocial and neutral interactions among young adults, considering their retrospective experiences of victimization. Surprisingly, individuals who had experienced victimization, as assessed through self-report questionnaires, diverted their early attention away from bullying and cyberbullying videos compared to the prosocial and neutral ones, likely to avoid triggering negative emotional responses based on past experiences. However, while the study merits acknowledgement for examining the influence of victimization on attentional patterns, its primary focus was measuring differences in attention across various types of interactions rather than differentiating attentional responses to specific social cues within bullying scenarios.

A subsequent study by Troop-Gordon et al. (2019) took a step further and analyzed differences among adolescents in encoding cues from bullying video clips featuring characters portraying the roles of bully, victim, pro-bully, defender, and bystander. They found that students with high levels of victimization and aggressiveness in their daily lives allocated more attention to observing the bully. Additionally, regardless of the level of victimization, aggressiveness exhibited a negative correlation with attention toward the victim.

Following these interesting findings, McConnell and Troop-Gordon (2021) investigated whether differences in daily life victimization and encoding social cues could be linked to different responses to victimization and coping strategies. They found that students who experienced high levels of victimization and paid more attention to the bully were more likely to engage in retaliatory behaviors, compared to those who only experienced high levels of victimization without a heightened focus on the bully.

Overall, these studies offer compelling insights into the complexities of attention in bullying situations, underscoring the necessity for additional investigation in this less-explored area of research. Further studies are essential to deepen our understanding of how individual experiences of bullying can shape attentional responses. This addresses a crucial aspect that still lacks comprehensive exploration.

## Aim

The primary objective of this study was to explore the attentional patterns of students engaged as victims, bullies, bully-victims, or not involved while observing bullying drawings with different portraits acting the role of the bully, the victim, the pro-bully, the defender and the bystander. Gaining insight into how these different groups pay attention to social cues during bullying interactions may be crucial for comprehending the interpretation of bullying episodes and subsequent behaviors (Horsley et al., 2010; Laue et al., 2018). Indeed, as underlined in other contexts (e.g., Wadlinger & Isaacowitz, 2011) how individuals direct their attention can significantly shape their emotional experiences and behavioral responses. This understanding is especially valuable for the understudied yet high-risk group of bully-victim students (Gini et al., 2021).

Thus, drawing upon the limited research that has explored attentional patterns with eye-tracking technology and building on the existing literature that investigated the SIP by mainly using scenarios and vignettes, we expected that aggressive children (bullies and bully-victims) would demonstrate a higher tendency to direct their attention towards hostile cues (the bully and the pro-bully portraits) compared to students who were not involved in bullying. By contrast, we expected two potential alternative attentional patterns for victims: on the one hand, drawing on the work of Caravita et al. (2016) and Ziv et al. (2013), we hypothesized that victims might avoid watching the core of the aggression scene, possibly directing their attention towards less threatening cues (e.g., the defender, the bystander portraits). On the other hand, considering the work of McConnell and Troop-Gordon (2021) and previous research on SIP and bullying (Camodeca et al., 2003), an alternative hypothesis is that victims might display heightened sensitivity to hostile cues due to their past negative experiences.

## Method

### Participants

The study involved four classes from two lower secondary public schools in the Emilia-Romagna region (North Italy). Every family had provided parental consent for their child's participation. However, on the day of the study, very few students (less than 5%) were absent, resulting in a total of 80 participants included in the research.

Students were excluded if at least one index (visit count, fixation count, and fixation duration, for the definition of the attentional indexes, please refer to the paragraph titled "Eye-tracker and Attentional Indexes”) deviated by ±2 standard deviations. In this regard, due to their potential impact on the study's findings, we removed seven students from the analysis to ensure the validity of our results and minimize the potential confounding factors associated with possible task comprehension difficulties, attentional issues, or other related variables.

The ages of the students ranged from 10 to 12 years, with a mean age of 11.18 years and a standard deviation of 0.30. The majority of the participants identified as Italian, with only two students not reporting Italian nationality.

### Tools

#### Stimuli

According to the method used by Menabò et al. (2023), the eye-tracking experiment included nine drawings depicting various forms of bullying. Three drawings represented physical, verbal, and relational bullying, and in each vignette, all the roles (bully, victim, pro-bully bystander, defender, bystander) were portrayed. To ensure gender balance, each type of bullying vignette featured male portraits in one, female portraits in another, and a third described a mixed-gender scenario. Importantly, each drawing within the same type of bullying depicted distinct content. For example, in the case of physical bullying, the male scene illustrated a child pushing another, the female scene portrayed the bully tripping her victim, and in the mixed scene, the bullies threw paper balls at the victim. The vignettes aimed to capture experiences that are commonly encountered by young people, such as physical acts like pushing and relational behaviors like exclusion from a game. The selection of scenes was guided by recommendations from various authors (e.g., Patchin & Hinduja, 2006; Troop-Gordon et al., 2019) to ensure the depiction of all characteristics typically associated with the three forms of bullying. For a detailed and comprehensive description of the drawings, please refer to Menabò et al. (2023).

#### Eye-tracker and Attentional Indexes

The eye movements of the participants were recorded using the Tobii Pro X2/60 eye-tracking device, which sampled gaze location at a rate of 60 Hz. In this study, each different role portrayed in the scenes was considered a separate “Area Of Interest” (AOI). An AOI represents a chosen portion of selected regions within a stimulus, enabling the extraction of specific attentional indexes for those locations.

An attentional index refers to a measure or indicator used to assess the allocation, distribution, or intensity of attentional resources in various cognitive and perceptual processes (e.g., Linz et al., 2016). In the present study, we considered fixation count, visit count, and total fixation duration. More specifically, fixation count represents the instances when a participant's gaze comes to a stop on a specific role (AOI) in the vignettes. Visit count indicates the number of times a participant transitions in and out of a specific role (AOI) in the vignette. Finally, the Total Fixation Duration (measured in seconds) is the total time spent on a specific role (AOI), providing an overall measure of sustained attention (for further information, see Menabò et al., 2023).

Consequently, each vignette presented five distinct AOIs, corresponding to the different portraits depicted in the scene (i.e., bully, victim, pro-bully, defender, bystander). The drawings were displayed to the students on a 19-inch monitor with a resolution of 1600 × 900 pixels. To ensure optimal configuration for children and pre-adolescents, a recommended 5-point calibration procedure, as outlined by Dys (2019), was employed. During calibration, the eye-tracker memorized the unique characteristics of each participant's eyes and accurately calculated the direction of their gaze on the surface of the screen. This process aimed to enhance the accuracy and reliability of the eye-tracking data collected during the experiment.

#### Bullying Experiences

To assess bullying experiences the Italian version of the *“European Bullying Intervention Project Questionnaire”* (EBIPQ, Brighi et al., 2012) was used. It comprises 14 items, 7 for victimization subscale and 7 for perpetration subscale, including specific behaviors such as direct physical abuse, indirect abuse, verbal abuse, and social exclusion in the last two months. It is based on a 5-point frequency scale (0 = no; 1 = yes, once or twice; 2 = yes, once or twice a month; 3 yes, about once a week; 4 = yes, more than once a week). The questionnaire demonstrated good overall reliability (Cronbach's α = 0.78). Using data from the questionnaire, participants were categorized into four groups: victims, bullies, bully-victims, or those not involved, according to their experiences. We applied the role classification method developed by Guarini et al. (2020), whereby students with scores between 0 and 1 in both victimization and perpetration sub-scales were categorized as “not involved”; those with a score of 2 or higher in the victimization scale and between 0 and 1 in the perpetration scale were labeled as “victims.” Participants with a score of 2 or higher on the perpetration scale and between 0 and 1 on the victimization scale were classified as “bullies.” Lastly, participants with a score of 2 or higher on both scales were categorized as “bully-victims”.

### Procedure

The first step involved students participating in the experiment with the eye-tracker. The first author conducted the experiment with the assistance of an expert psychologist in bullying in a dedicated room at the students’ schools. Students were invited to sit at a table and positioned in front of the screen and informed that their eye movements would be recorded as they watched vignettes related to bullying.

To ensure accurate gaze tracking, the eye-tracker was calibrated and validated, guaranteeing a gaze position accuracy of 0.50 degrees or better. The students had control over when they wanted to proceed to the next image by pressing the right arrow key on the keyboard. The entire experiment took approximately 10 minutes for each participant to complete, and no other questions were asked. A week later, the students completed an online questionnaire on Qualtrics that included demographic information and a scale measuring their experiences with bullying, both as victims and perpetrators. To ensure the correspondence between the two data sets (the eye-tracker experiment and the questionnaire), every participant was invited to choose a nickname at the beginning of the eye-tracker task. While we suggested using the first three letters of a parent or caregiver's name and with their class section, the final decision rested with the student. This self-selected nickname was also used by the participants for the questionnaire.

Before we started collecting data, we informed students that participation was voluntary and confidential. We specified to them that participation was purely for research purposes and that they could withdraw anytime without any negative consequence.

### Ethics

The study protocol met the ethical guidelines for the protection of human participants, including adherence to the legal requirements of Italy, and received formal approval by the Bioethics Committee of the University of Bologna. The parents of the children provided their informed written consent for participation in the study, data analysis and anonymous data publication. No economic incentives were provided to parents or students.

### Statistical Analysis

As only one student could be classified as a bully, due to the lack of representative data for this group, we decided to exclude this student from the analysis, resulting in a final sample size of 72 students. Thus, 21 (29%) students were classified as not involved, 31 (43%) as victims, and 20 (28%) as bully-victims (see Table A1 in Appendix A for gender distribution within each category). Before analyzing attentional differences in each role portrayed, we first assessed the total attentional indexes considering all the portrayed roles together. This assessment aimed to ensure that any potential differences observed among the groups (victims, bully-victims and not involved) were not solely attributed to variations in overall attention and to create a fair basis for comparing the students’ groups. We used the Kruskal-Wallis test in SPSS v25, revealing no significant differences among the students classified as not involved, victims, and bully-victims in terms of fixation count (*H* = 5.262, *p* = .07), visit duration (*H* = 3.957, *p* = .138) and total fixation duration (*H* = 4.567, *p* = .102). Given that there was a violation of the assumption of normality in all measures (Shapiro-Wilk: p < .001 for each attentional index for each portrait), we chose to conduct non-parametric analyses. We therefore conducted the Kruskal-Wallis test to examine differences in attentional indexes among the three groups for each portrayed role. In cases where significant differences were detected, pairwise comparisons were conducted.

## Results

Students classified as victims or bully-victims showed noticeable differences in fixation count scores (Table 1) for the portraits of the bully (*H* = 6.070, *p* = .048) and the pro-bully (*H* = 6.729, *p* = .035). However, no significant effects were observed for the portraits of the victim (*H* = 2.315*, p* = .310), defender (*H* = 4.078, *p* = .130), and bystander (*H* = 3.755, *p* = .153). Regarding the significant difference for the bully portrait, post-hoc comparisons (*H* = -14.494, *p* = .047) revealed that bully-victims (*M_rank_* = 46.08) had higher fixation counts than victims (*M_rank_* = 31.58), while no differences were found among these groups and students not involved (*M*_rank_ = 34.64). Similarly, for the pro-bully portrait post hoc comparisons indicated a significant difference (*H* = -14.298, *p* = .042) with higher fixation counts in bully-victims (*M*_rank_ = 46.48) compared to the victims (*M_rank_* = 32.18), while students not involved did not differ from the other groups (*M_rank_* = 33.38).

**Table 1:** Fixation Count of Students with Different Bullying Experiences.

		Victim (A)		Students bully-victim (B)		Not involved (C)		*χ^2^*(2)	*p*	Post-hoc comparison
		N	Mean of rank	N	Mean of rank	N	Mean of rank			
	Bully	31	31.58	20	46.08	21	34.64	6.070	.048	B>A
**Portraits**	Victim	31	33.03	20	44.50	21	34.00	2..315	.310	/
	Pro-bully	31	32.18	20	46.48	21	33.38	6.729	.035	B>A
	Defender	31	32.06	20	43.52	21	36.36	4.078.	.130	/
	Bystander	31	34.90	20	42.42	21	33.21	3.755	.153	/

*Note*. The table presents variations in the Fixation Count among students categorized as victims, bully-victims, or not-involved in observing bully, victim, pro-bully, defender and bystander portraits.

Regarding visit counts (Table 2), a significant effect was again observed among the three groups (victims, bully-victims and not involved) in relation to the exploration of the bully (*H* = 6.847, *p* = .033) and pro-bully portraits (*H* = 7.709, *p* = .021). Conversely, no significant effects were found for the victim (*H* = 5.556, *p* = .062), the defender (*H* = 5.558, *p* = .061), and the bystander (*H* = 2.778, *p* = .249) portraits. Post hoc comparisons for the bully portrait revealed a significant difference (*H* = -15.662, *p* = .027) with higher scores in bully-victims (*M_rank_* = 46.27) than victims (*M_rank_* = 30.61), while not involved students did not differ from the other groups (*M_rank_* = 35.88). Similarly, for the pro-bully portrait, post hoc comparisons indicated a significant difference (*H* = -15.806, *p* = .020) between students categorized as bully-victims (*M_rank_* = 47.00), who presented higher scores, and victims (M*_rank_*= 31.19), while no differences emerged for students not involved (*M_rank_* = 34.33).

**Table 2:** Visit Count of Students’ with Different Bullying Experiences.

		Victim (A)		Students bully-victim (B)		Not involved (C)		*χ^2^*(2)	*p*	Post-hoc comparison
		N	Mean of rank	N	Mean of rank	N	Mean of rank			
	Bully	31	30.61	20	46.27	21	35.88	6.847	.033	B>A
**Portraits**	Victim	31	32.37	20	45.83	21	33.71	5.556	.062	/
	Pro-bully	31	31.19	20	47.00	21	34.33	7.709	.021	B>A
	Defender	31	30.95	20	44.90	21	36.69	5.558	.061	/
	Bystander	31	33.82	20	43.10	21	34.17	2.778	.249	/

*Note.* The table presents variations in the Visit Count among students categorized as victims, bully-victims, or not involved in observing bully, victim, pro-bully, defender, and bystander portraits.

Regarding the total fixation duration (Table 3), no significant differences emerged.

**Table 3:** Total Fixation Duration of Students’ with Different Bullying Experiences.

		Victim (A)		Students Bully-victim (B)		Not Involved (C)		*χ^2^*(2)	*p*	Post-hoc comparison
		N	Mean of rank	N	Mean of rank	N	Mean of rank			
	Bully	31	31.97	20	44.73	21	35.36	4.61	.100	/
**Portraits**	Victim	31	31.03	20	44.50	21	36.95	5.05	.080	/
	Pro-bully	31	32.13	20	44.83	21	35.02	4.91	.086	/
	Defender	31	30.82	20	43.15	21	38.55	4.65	.098	/
	Bystander	31	34.68	20	41.58	21	34.36	1.65	.438	/

*Note.* The table presents variations in the Total Fixation Duration among students categorized as victims, bully-victims, or not-involved in observing bully, victim, pro-bully, defender and bystander portraits.

## Discussion

The current study examined attentional patterns of victims, bully-victims, and not involved students while observing bullying scenarios. Our findings revealed an association between different experiences in bullying involvement in daily life and distinct attention allocations, confirming differences at the first stage of the SIP. However, it is interesting to note that these distinctions did not arise between bully-victims, victims and not involved students, but only between bully-victims and victims. Indeed, bully-victims demonstrated a higher level of attention compared to victims when observing portraits engaging in aggressive actions. This was evident through more fixations and visits to the portraits of the bully and pro-bully. The high number of fixations revealed a detailed exploration of these portraits, while high scores in visiting counts suggested the need to compare these portraits with the other characters included in the scene.

While previous studies have already described students involved as bully-victims as a specific subgroup of students, reporting the poorest psychosocial health compared with bullies, victims, and not-involved students (Kumpulainen & Räsänen, 2000; Stein, Dukes, & Warren, 2006), our study contributes to addressing a notable gap in our comprehension, particularly concerning the early stage of the SIP (Horsley et al., 2010). Interestingly, when encoding social cues was evaluated by traditional methods based on the recall of vignettes and scenarios, no differences were observed among students experiencing different roles in bullying daily lives (e.g., Camodeca et al., 2005). By contrast, when encoding social cues were evaluated in assessing attention allocation, some differences emerged. Indeed, our results align with the research of Troop-Gordon et al. (2019), who found an association between victimization, aggressiveness, and attention in observing the different roles. This connection underscores the relevance of our findings and the critical need to understand the complexities of attentional processes in the context of bullying.

To gain a comprehensive understanding of the different patterns of attention allocation described for bully-victims compared to pure victims, it could be helpful to consider previous research focusing on the levels of reactive and proactive aggression in bully-victims. Reactive aggression is conventionally characterized as impulsive, thoughtless, and a responsive reaction to provocation (Schwartz, Dodge, Pettit, & Bates, 1997). In contrast, proactive aggression is delineated as purposeful behavior (Unnever, 2005). Initially, research suggested that bully-victims could exhibit a higher degree of reactive aggression compared to victims and bullies (e.g., Ragatz, Anderson, Fremouw, & Schwartz, 2011). Nevertheless, other studies indicated that bully-victims also displayed a noteworthy level of proactive aggression, surpassing even that of bullies (e.g., Salmivalli & Nieminen, 2002; Camodeca & Goossens, 2005). In an interesting study by Runions and colleagues (2013), four distinct motives for perpetration—recreation, reward, revenge, and rage—were analyzed among bullies, victims, bully-victims, and those not involved. Bully-victims reported substantially elevated levels across each motive in comparison to victims confirming both reactive and proactive aggressions.

Given these premises, the first explanatory hypothesis of higher attentional scores of bully-victims than victims in observing portrayed roles that actioned aggression, is grounded in the observation that bully-victims tend to exhibit higher levels of reactive aggression. In this regard bully-victims may pay more attention to bully and pro-bully portraits compared to the victims because they feel threatened and this could influence emotional responses and activate dysfunctional coping behaviors (Andreou, 2001; Bijttebier & Vertommen, 1998; Crick & Dodge, 1994). Previous literature has shown that bully-victims may exhibit cognitive processing biases and emotional regulation difficulties and that these factors, in turn, may lead to aggressive behaviors when they perceive threats (Rosen, Milich, & Harris, 2009). For example, victims with anger management difficulties are likely to increase physiological reactions, potentially contributing to an escalation of aggressive behaviors (Kaynak, Lepore, Kliewer, & Jaggi, 2015). Our findings suggest that their heightened attention to threat signals may trigger a cascade of emotional and aggressive behavioral responses, ultimately negatively influencing their experiences and interactions within the social environment.

Alternatively, the second explanatory hypothesis may suggest that bully-victims may pay more attention than victims to bully and pro-bully portraits due to the perceived social status or power associated with these roles. For this reason, bully-victims may observe these portraits with great focus, considering them as examples of achieving desired outcomes through intimidation and proactive aggression. This aligns with research using eye-tracking technology, suggesting a consistent inclination among individuals to focus on roles associated with high status (Foulsham, Cheng, Tracy, Henrich, & Kingstone, 2010; see also the review Cheng et al., 2023 on eye-gaze and leadership).

While the first hypothesis highlights the potential heightened attention of bully-victims to threat cues as a signal of the preparation of an aggressive response, the second hypothesis draws attention to the bully and pro-bully portraits as perceived sources of social status and power, serving as examples of acted aggressions. These two perspectives can either stand as distinct alternatives or also intersect, as showed by the fact that bully-victims show both levels of reactive and proactive aggression. In this case, bully-victims may feel threatened while concurrently acknowledging the bully and the pro-bully as the figures with more power. This dual perspective might function as a coping or adaptation strategy, particularly if bully-victims aim to improve their social standing or address perceived threats in their social environment.

Concerning pure victims, previous research has suggested that pure victims are more likely to be submissive and withdrawn rather than exhibiting aggressive tendencies (Toblin, Schwartz, Hopmeyer Gorman, & Abou-ezzeddine, 2005). According to the SIP framework, victims may tend to avoid social situations due to their anticipation of hostility, as observed in Ziv et al. (2013). In addition, Caravita et al. (2016) reported that young adults with prior victimization experiences displayed less attention to scenes characterized by aggression, probably to protect themselves from triggering negative emotions. However, it is important to note that their research examined attention to scenes in a general sense rather than focusing on individual roles while our results show that victims do not have different total fixation times on general scenes but show differences in times spent on specific roles.

To the best of our knowledge, our study is the first to provide evidence supporting the idea that victims may look away from threatening stimuli in a scene compared to the bully-victims. This observation is grounded in an essential state: the automatic regulation of visual attention is crucial to preventing excessive arousal and managing emotional reactions, representing the “first line of defense” against potential threats (Wadlinger & Isaacowitz, 2011, p. 3). In this regard, although not directly related to bullying, previous studies have shown that children with low levels of perceived control may disengage their attention in response to fear-inducing stimuli (Vasey, El-Hag, & Daleiden, 1996). Indeed, while children with high levels of perceived control tend to maintain focused attention and seek additional information, children with low control may unconsciously prioritize managing their emotional reactions to potential threats (Vasey et al., 1996). This suggests that individuals' attentional patterns are correlated with their perceptions of their ability to cope with and control threatening situations.

In conclusion, our study represents a significant contribution to the field as it is the first to provide evidence of distinct attentional patterns among victims and bully-victims. These findings hold significant implications for our understanding of bullying dynamics and the development of effective interventions. In this regard,we emphasize that interventions should consider the diverse behavioral patterns of attention that can either fuel aggressive behaviors or lead to avoidance responses. How visual attention patterns can be related to emotions and subsequent behaviors should be a crucial component of prevention and intervention strategies, as highlighted by Wadlinger and Isaacowitz (2011). Indeed, we argue that by recognizing the intricate interplay between attentional patterns and their role in exacerbating or alleviating victimization, interventions can be refined to target the root causes of bullying more effectively.

To comprehensively address this complex issue, educational and developmental psychology fields need to adopt an interdisciplinary approach that integrates methodologies from various disciplines. By exploring the biological, psychological, and sociocultural factors that contribute to attentional patterns, researchers can gain deeper insights into the mechanisms underlying bullying and, collaborating closely with all relevant stakeholders, develop more precise and targeted interventions.

## Limitations and Future Research

Although the present research represents an important step in the study of attention in bullying, limitations should be considered. The first limitation is the absence of pure bullies in our sample. It is possible that individuals who engage in aggressive behavior may also report victimization experiences to mitigate the negative social perceptions of being solely identified as a bully. Nevertheless, it is important to note that previous studies have shown that half of the students reporting being a bully also report victimization (Haynie et al., 2001; Solberg, Olweus, & Endresen, 2007). This suggests that individuals engaging in aggressive behavior may have complex experiences making it challenging to categorize them solely as bullies. In this regard, it could be worth including an assessment of reactive and proactive aggression to better understand the attentional patterns of bully-victims.

The second limitation, connected to the previous one, pertains to the categorization we employed (victims, bully-victims, and not-involved) that presents two noteworthy aspects of attention. Firstly, students not involved can be defenders, pro-bullies and bystanders; secondly, we treated roles as static entities, whereas individuals' roles in bullying can significantly change across diverse contexts and situations (Belacchi, Altoè, & Caravita, 2023). A student engaging in aggression in one episode might assume a victim role in another or even adopt a bystander position in yet another context. To address these complexities, future research should consider incorporating additional methodologies, such as multiple behavioral observations or peer nominations. This approach would not only enhance the identification of bullies, bully-victims, and victims but also shed light on the roles of defenders, pro-bullies, and others from various perspectives.

The third limitation pertains to the stimuli employed in our study (see Menabò et al., 2023). Indeed, despite our efforts to distribute portraits across various sections of the scenes in different vignettes, achieving complete balance in this regard proved unfeasible. In addition, even if using human actors in visual stimuli can enhance ecological validity, we opted to utilize comic drawings to address potential social biases inherent in human interactions, such as those related to gender, race, or physical appearance (Riby & Hancock, 2009). This choice was made to mitigate the influence of these socially demanding factors, as shown in the Fig. 1 provided in Appendix A.

The final limitation was the size of our sample. Although it aligns with previous eye-tracking studies (Troop-Gordon et al., 2019) and is even larger in some cases (Horsley et al., 2010; Laue et al., 2018), it has led to relatively small groups representing distinct bullying roles. In addition, our study did not specifically control if students with Special Education Needs (e.g., students with ADHD) were included in the sample. Nonetheless, we excluded students with outlier scores in attentional indexes, and we did not observe differences in the total time spent observing scenes between bully-victims, victims and not-involved. Future research should include a larger sample of students to enhance the reliability of our results.

## Conclusions

In summary, our study revealed distinct attentional patterns between bully-victims and victims, emphasizing the critical need for prevention and intervention programs to adopt a comprehensive approach that also considers the underlying attentional processes.

Regarding bully-victims, if their attention to bullies and pro-bullies is due to the need to control threatening stimuli, it could be beneficial to propose activities to regulate emotional responses and foster more adaptive coping strategies. In this regard, social emotional learning (SEL) programs can play a pivotal role. SEL focuses on developing skills such as self-awareness, self-management, social awareness and responsible decision-making (Llorent, Diaz-Chaves, Zych, Twardowska-Staszek, & Marín-López, 2021).

Conversely, a different intervention approach is warranted if their attention to them aligns with a sense of attraction or positive reward. In such cases, interventions should focus on challenging and reshaping the peer group norms and societal roles that contribute to the perception of bullies as popular. These interventions need to introduce and reinforce alternative popularity models based on positive, respectful, and inclusive behaviors rather than aggression or dominance. To achieve this, changing the normative rules within these social contexts is essential. In this vein, Italy has made significant efforts at the national level in recent years, implementing many initiatives in schools (e.g.,“General guidelines and national actions to prevent and contrast school-bullying”) aimed at preventing and contrasting bullying (Ministry of Education and Merit, 2022).

In cases where both threat perception and attraction to the bully coexist within bully-victims, it is essential to recognize the intricate nature of their experiences and motivations. A multifaceted intervention approach should be employed to address this complexity effectively, including elements from threat perception and attraction mitigation strategies. These efforts could play a pivotal role in reshaping their perceptions and ultimately guiding them toward more constructive and empathetic behaviors.

Victims may benefit more from interventions that empower them with effective coping strategies, fostering a sense of control over their situations and behaviors. A key part of these interventions is focusing on building resilience. Resilience is important because it helps individuals recover from difficult experiences and maintain their mental health. Indeed, by enhancing resilience, victims learn to adapt to challenges, lessen the effects of stress, and become stronger and more capable of handling future adversities (Fang, Lu, & Che, 2022).

To conclude, by acknowledging the multifaceted nature of bullying dynamics and recognizing the pivotal role of attention in shaping behavioral responses, we take a significant step toward addressing the root causes and consequences of bullying. This, in turn, can lead to healthier, harmonious social interactions for everyone involved.

## Declaration of competing interest

All authors declare that they have no conflicts of interest.

## Figures and Tables

**Table A1:** Gender Distribution of Each Experienced Role.

	Victim		Bully-victim		Not involved	
	(A)		(B)		(C)	
	N	%	N	%	N	%
Males	11	52%	16	46.2	10	35.8
				7		8
Females	10	48%	15	45.8	10	33.7
				3		1

**Picture 1:**
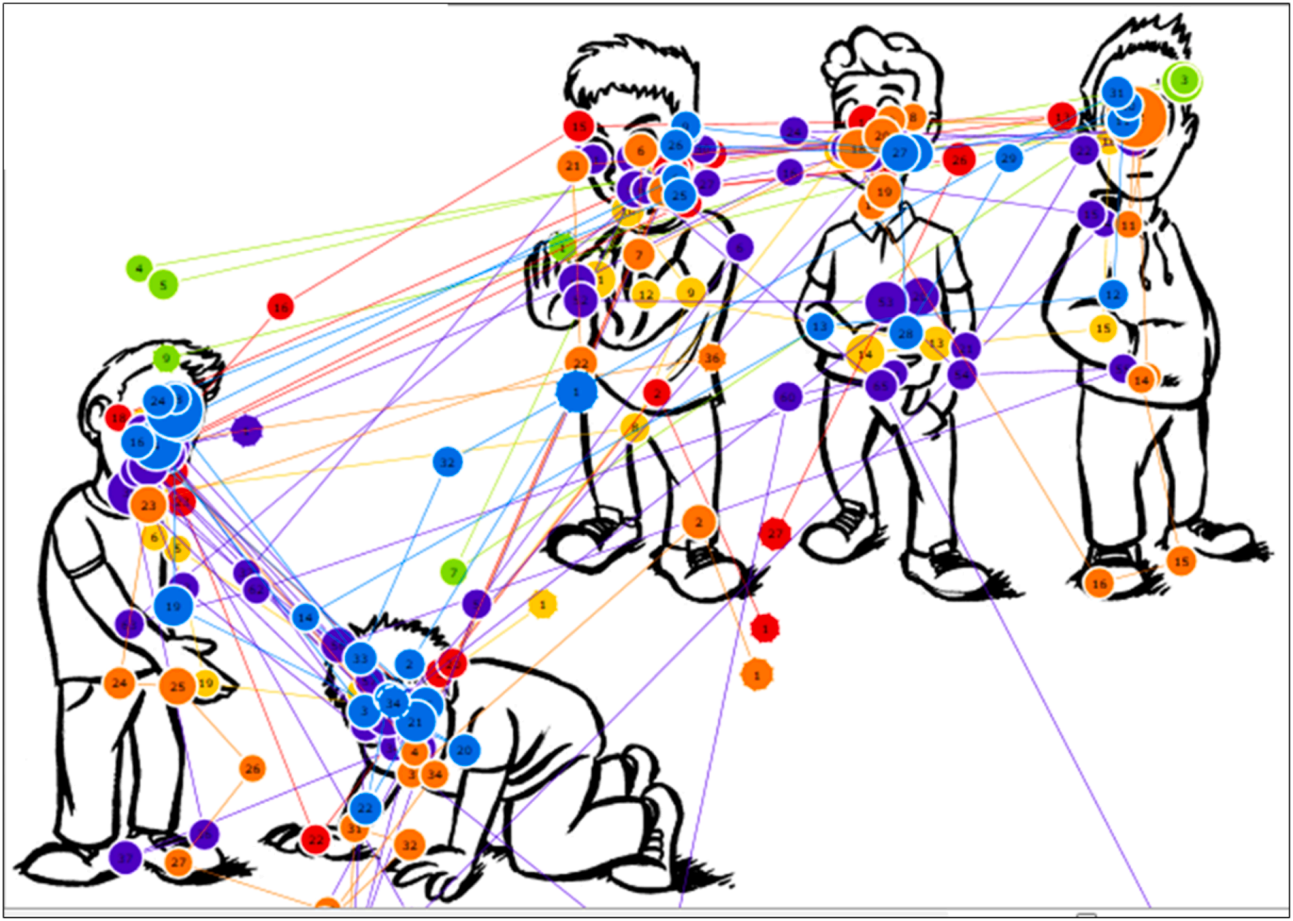
Physical Bullying.

## APPENDIX A

The image depicts the visual portrayal of the eye movements of 10 students while they observed a scenario of physical bullying. From the left, the defender is shown assisting the victim, who has fallen to the ground and is now being helped up. The bully, with hands still outstretched, is depicted alongside the bystander, observing the scene without intervening. The choice to include the eye movements of 10 participants was made to prevent excessive overlap and uphold the vignette's clarity.
